# Supplementary material for: The genome sequence of a tephritid fly, Philophylla caesio (Harris, 1780)
Source: Wellcome Open Res. 2025 Jul 9;10:338. [Version 1] doi: 10.12688/wellcomeopenres.24331.1 (PMC12759287; doi:10.12688/wellcomeopenres.24331.1)
Supplement: Peer review history

# Reviewer report: Reviewer response for version 1

**DOI:** 10.21956/wellcomeopenres.26832.r142825

**Published:** 2026-01-02

**Authors:** Danilo Trabudo do Amaral

**Affiliations:** 1 Universidade Federal de Sao Carlos, São Carlos, State of São Paulo, Brazil

This manuscript presents a high-quality, chromosomally resolved genome assembly of Philophylla caesio, generated using a robust and well-established PacBio HiFi and Hi-C pipeline. The assembly quality metrics (BUSCO, Merqury, BlobToolKit) indicate that the genome meets current reference standards and is suitable for downstream analyses.

 As a minor but important suggestion, I recommend assessing the quality of the annotated gene set by running BUSCO on the predicted protein sequences. Given that no RNA-seq data were generated, reporting BUSCO completeness for the annotation would provide valuable information on the reliability of the gene predictions and further strengthen the utility of this dataset.

Are sufficient details of methods and materials provided to allow replication by others?

Yes

Is the rationale for creating the dataset(s) clearly described?

Yes

Are the datasets clearly presented in a useable and accessible format?

Yes

Are the protocols appropriate and is the work technically sound?

Yes

Reviewer Expertise:

Bioinformatics

I confirm that I have read this submission and believe that I have an appropriate level of expertise to confirm that it is of an acceptable scientific standard.

# Reviewer report: Reviewer response for version 1

**DOI:** 10.21956/wellcomeopenres.26832.r128244

**Published:** 2025-08-21

**Authors:** Panagiotis Ioannidis

**Affiliations:** 1 Foundation for Research & Technology - Hellas, Crete, Greece

This paper reports the sequencing and assembly of the genome of a tephritid fly.

 The methodology used for assembling this genome is the standard one used by all DToL projects (PacBio + Hi-C). Thus, the assembly produced is of adequate quality for any downstream analyses, as it is witnessed by the standard QC analyses carried out here (BUSCO, MerquryFK).

 I do have one rather important comment. A gene set was predicted for this genome assembly which resulted in ~32K genes. However, without including the BUSCO score for this gene set one cannot know the quality (and thus suitability) of this gene set for downstream analyses. And this is especially true  for the current species, since no RNAseq data were generated, which could help improve the accuracy of gene prediction. In other words, if you feel "obliged" to run BUSCO for your genome assembly, then you should also feel obliged to run BUSCO for your gene set (should you generated one).

Are sufficient details of methods and materials provided to allow replication by others?

Yes

Is the rationale for creating the dataset(s) clearly described?

Yes

Are the datasets clearly presented in a useable and accessible format?

Yes

Are the protocols appropriate and is the work technically sound?

Yes

Reviewer Expertise:

bioinformatics, insect genomics

I confirm that I have read this submission and believe that I have an appropriate level of expertise to confirm that it is of an acceptable scientific standard.
